# Rationale and study design of ViPS – variable pressure support for weaning from mechanical ventilation: study protocol for an international multicenter randomized controlled open trial

**DOI:** 10.1186/1745-6215-14-363

**Published:** 2013-10-31

**Authors:** Thomas Kiss, Andreas Güldner, Thomas Bluth, Christopher Uhlig, Peter Markus Spieth, Klaus Markstaller, Roman Ullrich, Samir Jaber, Jose Alberto Santos, Jordi Mancebo, Luigi Camporota, Richard Beale, Guilherme Schettino, Felipe Saddy, Immaculada Vallverdú, Bärbel Wiedemann, Thea Koch, Marcus Josephus Schultz, Paolo Pelosi, Marcelo Gama de Abreu

**Affiliations:** 1 Department of Anesthesiology and Intensive Care Medicine, Pulmonary Engineering Group, University Hospital Dresden, Technische Universität Dresden, Dresden, Germany; 2 Department of Anesthesiology, Intensive Care Medicine and Pain Therapy, Medical University of Vienna, Vienna, Austria; 3 Department of Critical Care Medicine and Anesthesiology, Saint Eloi University Hospital and Montpellier School of Medicine, Montpellier, France; 4 Hospital de la Santa Creu i Sant Pau, Barcelona, Spain; 5 Department of Adult Critical Care, St Thomas’ Hospital, Guy’s and St Thomas’ NHS Foundation Trust, London, United Kingdom; 6 Intensive Care Unit, Hospital Sírio-Libânes, São Paulo, Brazil; 7 Hospital Copa D’Or, Rio de Janeiro, Brazil; 8 Hospital Sant Joan, Reus, Spain; 9 Institute for Medical Informatics and Biometry, Technische Universität Dresden, Dresden, Germany; 10 Department of Intensive Care Medicine, Academic Medical Center at the University of Amsterdam, Amsterdam, The Netherlands; 11 Department of Surgical Sciences and Integrated Diagnostics, IRCCS San Martino Hospital IST, University of Genoa, Genoa, Italy

**Keywords:** Mechanical ventilation, Weaning, Pressure support ventilation, Variable ventilation, Intensive care unit, Critical care

## Abstract

**Background:**

In pressure support ventilation (PSV), a non-variable level of pressure support is delivered by the ventilator when triggered by the patient. In contrast, variable PSV delivers a level of pressure support that varies in a random fashion, introducing more physiological variability to the respiratory pattern. Experimental studies show that variable PSV improves gas exchange, reduces lung inflammation and the mean pressure support, compared to non-variable PSV. Thus, it can theoretically shorten weaning from the mechanical ventilator.

**Methods/design:**

The ViPS (variable pressure support) trial is an international investigator-initiated multicenter randomized controlled open trial comparing variable vs. non-variable PSV. Adult patients on controlled mechanical ventilation for more than 24 hours who are ready to be weaned are eligible for the study. The randomization sequence is blocked per center and performed using a web-based platform. Patients are randomly assigned to one of the two groups: variable PSV or non-variable PSV. In non-variable PSV, breath-by-breath pressure support is kept constant and targeted to achieve a tidal volume of 6 to 8 ml/kg. In variable PSV, the mean pressure support level over a specific time period is targeted at the same mean tidal volume as non-variable PSV, but individual levels vary randomly breath-by-breath. The primary endpoint of the trial is the time to successful weaning, defined as the time from randomization to successful extubation.

**Discussion:**

ViPS is the first randomized controlled trial investigating whether variable, compared to non-variable PSV, shortens the duration of weaning from mechanical ventilation in a mixed population of critically ill patients. This trial aims to determine the role of variable PSV in the intensive care unit.

**Trial registration:**

clinicaltrials.gov NCT01769053

## Background

Around 40% of the total duration of mechanical ventilation is spent in the weaning process [1-3]. Both prolonged mechanical ventilation, as well as premature extubation, are associated with severe complications. For example, the risk of developing pneumonia increases with the duration of mechanical ventilation [4,5] and with re-intubation [6]. More importantly, failed extubation is associated with an increase in the risk of death [7]. Therefore, delivery of mechanical ventilatory support for the shortest possible time is the state of the art in intensive care medicine [8].

Pressure support ventilation (PSV) is the commonest form of spontaneous assisted mechanical ventilation worldwide [3]. During weaning with PSV, the pressure support applied by the ventilator is reduced in a stepwise manner until a minimal level of support is achieved. During classical (non-variable) pressure support, each spontaneous breath is supported by a constant set level of pressure at the airways. Thus, breath-by-breath variability of tidal volume (*V*_*T*_) and respiratory rate (RR) is mainly determined by the patient’s respiratory drive. A low respiratory drive, due to underlying disease and/or deep sedation, may result in a respiratory pattern with minimal or no variability despite the spontaneous breathing.

Decreased variability in *V*_*T*_ and RR is associated with delayed weaning from mechanical ventilation in a mixed intensive care unit (ICU) population [9]. In patients recovering from sepsis, increased variability of *V*_*T*_ and RR is a reliable predictor of weaning success [10]. However, those studies were conducted retrospectively and, to our knowledge, no study has tested whether weaning from mechanical ventilation can be made shorter by increasing the variability of the tidal volumes using variable levels of pressure support. A possible reason for the lack of such a study might be because assisted modes of mechanical ventilation resulting in more variable respiratory patterns (for example, proportional assist ventilation and neurally adjusted ventilatory assist) depend on the intrinsic variability of the respiratory system. Another likely explanation is that a desired mean *V*_*T*_ is difficult to guarantee in closed-loop systems that sense the inspiratory effort, making comparisons of variable with non-variable assisted mechanical ventilation at a predefined mean *V*_*T*_ difficult.

In 2008, a new ventilation strategy termed variable (or noisy) PSV was introduced. Variable PSV is able to increase the variability of the respiratory pattern independent of the inspiratory effort [11]. Thereby, matching of *V*_*T*_ between variable and non-variable PSV can be easily accomplished. Preliminary observations suggest that variable PSV has the potential to reduce patient-ventilator asynchrony, but it is uncertain whether this is associated with a shorter duration of weaning from mechanical ventilation.

Since variable PSV can reduce the mean pressure support [12], it may lead to a faster reduction of pressure support and, therefore, a shorter weaning period than non-variable PSV. We describe the design of the first randomized controlled trial of variable PSV for weaning from mechanical ventilation in a mixed intensive care unit population.

## Methods/design

### Objectives and design

The ViPS trial is an international investigator-initiated multicenter randomized controlled open trial comparing variable vs. non-variable pressure support ventilation in patients receiving mechanical ventilation for more than 24 hours who are able to be weaned. The Ethics Committee of the Dresden University of Technology has approved the protocol of the trial. The ViPS trial is conducted in accordance with the declaration of Helsinki and was registered on 18 December 2012 as a clinical trial [13] with trial identification number NCT01769053.

**Figure 1:**
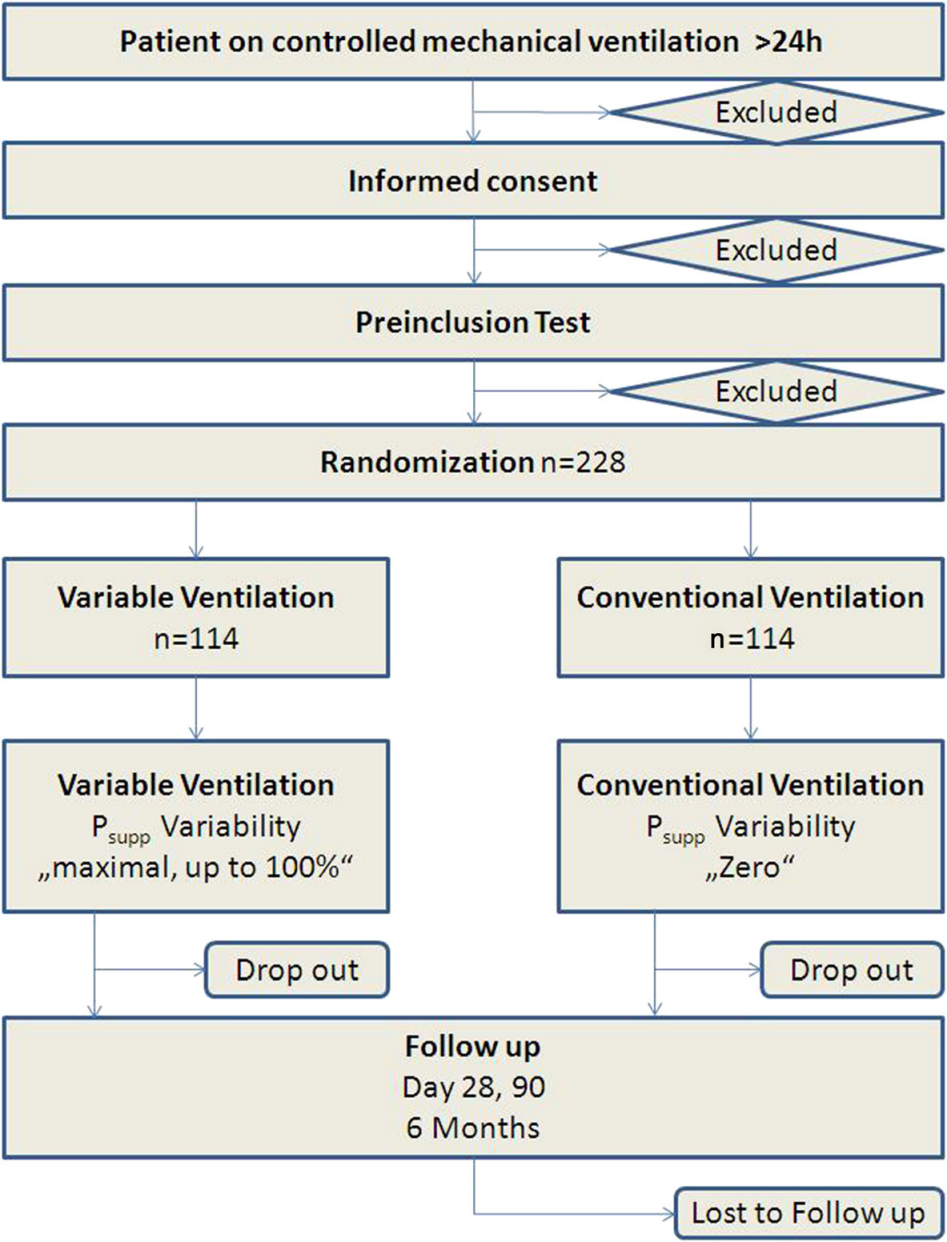
**CONSORT diagram of the trial.***P*_supp_ is the level of pressure support.

The objective of the study is to test whether variable compared to non-variable pressure support ventilation effects the duration of weaning from mechanical ventilation. The hypothesis of the ViPS trial is that variable pressure support ventilation will shorten the duration of weaning from mechanical ventilation compared to non-variable pressure support ventilation by two days. Figure 1 shows the consolidated standards of reporting trials (CONSORT) diagram of the ViPS trial.

### Patient screening

Consecutive intensive care unit patients who are on controlled mechanical ventilation for more than 24 hours are eligible for the trial. Local investigators screen patients admitted to ICU on a daily basis. All patients, their legal representative or an independent physician not involved with the study, depending on local regulations, are asked for signed informed consent, as required by the local ethics committee in accordance with the declaration of Helsinki.

### Inclusion and exclusion criteria

The inclusion criteria are the following: age ≥18 years; duration of controlled mechanical ventilation ≥24 h; temperature ≤39°C; hemoglobin ≥6 g/dl; ratio of arterial partial pressure of oxygen to inspiratory oxygen fraction PaO_2_/FiO_2_ ≥ 150 mmHg with positive end-expiratory pressure (PEEP) ≤16 cmH_2_O; ability of the patient to breathe spontaneously; availability of a mechanical ventilator with the capability for variable PSV and informed consent according to local regulations.

The following exclusion criteria have been defined: the patient has participated in another interventional trial within the last four weeks before enrolment in the ViPS trial; peripheral neurological disease associated with impairment of the respiratory pump; muscular disease associated with impairment of the respiratory pump; unstable thorax with paradoxical chest wall movement; planned surgery under general anesthesia within 72 hours; difficult airway or intubation; existing tracheotomy at ICU admission; expected survival <72 hours; home mechanical ventilation or on chronic oxygen therapy and suspected or confirmed pregnancy of the patient.

### Pre-inclusion test

Upon giving informed consent and after confirming agreement with the inclusion and exclusion criteria, a patient is monitored for spontaneous breathing activity. The transition from controlled to assisted ventilation is observed by a so-called pre-inclusion Test. After switching controlled mechanical ventilation to pressure support mode, every patient has to fulfill specific criteria for a period of one hour. This phase is to ensure that patients in both treatment groups show uniform stable spontaneous breathing activity. The time at which spontaneous breathing activity occurs will be recorded. This initial test can be interrupted and controlled mechanical ventilation resumed if the patient shows signs of hemodynamic or respiratory distress during the pre-inclusion test.

The pre-inclusion test is considered successfully passed if: temperature ≤39°C; PaO_2_/FiO2 ≥ 150 mmHg with PEEP ≤16 cmH_2_O; pH ≥ 7.30; respiratory rate ≤40/min; heart rate 40 to 130/min; systolic blood pressure >80 or <160 mmHg; a dose of noradrenaline or adrenaline <0.1 mcg/kg/min, dopamine or dobutamine <2 mcg/kg/min; no hemodynamically relevant acute cardiac arrhythmias occur.

If the patient fails the test criteria, the test will be repeated after patient recovery, at the treating physician’s discretion. Randomization only occurs after the patient passes the pre-inclusion test.

### Randomization

Patients will be randomized using a web-based secure-sockets-layer (SSL) encrypted platform with a randomization sequence blocked per center to yield approximately equal group sizes (block randomization, 1:1 ratio, mixed block sizes maximum block size eight). Patients will be randomly assigned to one of the two groups: variable pressure support ventilation or non-variable pressure support ventilation. The assigned ventilation mode has to be applied immediately after randomization.

### Intervention

During the study all patients will be ventilated using PSV. In non-variable PSV, pressure support is constant breath-by-breath, and set to a target tidal volume. In variable PSV, the mean pressure support is set to achieve the same desired *V*_*T*_, but single values will vary randomly breath-by-breath.

### Ventilatory settings for both groups

Pressure support is to a targeted mean tidal volume of 6 to 8 ml/kg; the maximal inspiratory pressure ≤40 cmH_2_O; the flow trigger is set at 2 l/min; inspiratory cycling-off at 25% of peak flow; PEEP and FiO_2_ for oxygen saturation SaO_2_ ≥92%, with PEEP ≥5 cmH_2_O.

The adjustment of pressure support until extubation follows these rules and is equal for both therapy groups: pressure support is gradually adjusted in decrements (or increments) of 0 to 5 cmH_2_O. PEEP is decreased in steps of 0 to 5 cmH_2_O; PEEP and FiO_2_ are adjusted to achieve a SaO_2_ ≥92%, with a PEEP ≥5 cmH_2_O.

In patients ventilated with non-variable PSV, the pressure support variability is set to zero. In patients ventilated with variable PSV, the pressure support variability is as high as possible (up to 100%), while not exceeding the maximal inspiratory pressure determined by the treating physician.

The pressure support delivered by the ventilator in a given breath (P_supp_(*i*)) is determined by *P*_supp_(*i*) = Δ*P*_supp_ ± percentage-of-pressure-variability (*i*) × *P*_supp_, where the percentage-of-pressure-variability (*i*) is selected randomly from the set range and follows a Gaussian curve. Accordingly, for any given period of time each patient will receive more frequently *P*_supp_(*i*) values equivalent to Δ*P*_supp_ than extreme values of *P*_supp_(*i*).

Patients will be ventilated with the assigned mode of mechanical ventilation until extubation. In case of interruption of the assigned ventilation mode, for example during patient transport, the assigned mechanical ventilation mode has to be resumed as soon as possible.

### Extubation criteria

As the ventilator settings are reduced in a stepwise manner to reach the extubation criteria, the patient’s condition is continuously monitored. As soon as the patient fulfills the following extubation criteria for a time period of at least 30 minutes, extubation is possible:

Richmond agitation and sedation scale (RASS) ≥ −3; behavioral pain scale (BPS) ≤5 or visual analogue scale (VAS) for pain ≤3; raising hands or legs against gravity; temperature ≥36°C and ≤39°C; ability to cough to clear secretions after deflating cuff; respiratory rate 8 to 30/min; PaO_2_/FiO_2_ ≥ 200 mmHg (≥150 mmHg in patients with chronic obstructive pulmonary disease); pressure support ≤8 cmH_2_O; PEEP ≤8 cmH_2_O; systolic blood pressure 80 to 160 mmHg; heart rate 40 to 130/min and no hemodynamically relevant arrhythmia.

If extubation has occurred and the patient needs to be re-intubated within 72 hours, the extubation is defined as not successful and the assigned study mechanical ventilation mode will be reinstituted as soon as possible. Successful extubation is defined as extubation lasting 72 hours.

Non-invasive ventilation (NIV) after extubation is allowed after extubation according to local practice. However, if a PEEP level >8 cmH_2_O, or pressure support >8 cmH_2_O is required for NIV, the extubation is considered not successful.

If the patient has to receive tracheotomy during the study period, assisted mechanical ventilation will be resumed as soon as possible via the tracheal cannula according to the assigned therapy group. In tracheotomized patients successful extubation is defined as patient separation from the ventilator without reconnection for <72 h after separation. If continuous positive airway pressure (CPAP) therapy via the tracheal cannula with PEEP >8 cmH_2_O or pressure support >8 cmH_2_O is required, the separation from the mechanical ventilator is considered not successful. The study-related intervention is finished on successful extubation. Patients will be observed until ICU and hospital discharge and contacted during the follow-up period.

### Baseline data

After screening the following demographic characteristics will be documented: patient’s age, sex, height, weight, predicted body weight, date and type of hospital admission, date and type of surgery, date of ICU admission and time of mechanical ventilation. If a patient meets the inclusion and exclusion criteria we record the following: acute physiology and chronic health evaluation (APACHE) II score and sequential organ failure assessment (SOFA) score. Before and after the pre-inclusion test we record the following: date and time of pre-inclusion test, ventilation mode before and after pre-inclusion test, minute ventilation, respiratory rate, tidal volume, peak airway pressure, mean airway pressure, PEEP, FiO_2_, PaO_2_, arterial partial pressure of carbon dioxide (PaCO_2_) and pH.

### Intervention period data

During the study intervention, parameters are recorded daily at the same hour (the time of assessment is set by the local trial coordinator) within two hours of tolerance: SOFA score, ventilator mode, minute ventilation, respiratory rate, tidal volume, peak airway pressure, mean airway pressure, level of pressure support, set level of variability of pressure support, PEEP, airway inspiratory occlusion pressure at 100 ms (P0.1), FiO_2_, PaO_2_, PaCO_2_, pH, number of changes of pressure support settings, PEEP settings and FiO_2_ settings per 24 h, heart rate, mean arterial pressure, temperature, medication received in the last 24 hours (including sedatives, analgesics, neuromuscular blocking agents, corticosteroids and catecholamines) and fluid balance for the last 24 hours (including crystalloids, colloids and blood products). Selected centers will record the variation of tidal volume, pressure support, respiratory rate, inspiratory and expiratory times, as this requires additional equipment not available to all centers.

Because of logistical and organizational factors not every patient can be extubated immediately upon fulfilment of the extubation criteria (for example, at night). Therefore, the date and time of fulfillment of the extubation criteria and when extubation was performed are recorded separately. Successful extubation is documented after 72 hours of monitoring as explained before. With every extubation the intensive care delirium screening checklist score is assessed.

The need for NIV, NIV CPAP level and NIV pressure support level, as well as the total number of NIV hours, will be recorded. Incidences of tracheostomy and self-extubation will be registered.

### Study dropouts

Participation in the trial is voluntary. A subject has the right to withdraw from the study at any time for any reason without any consequences for further medical treatment. Furthermore the investigator has the right to terminate the participation of any subject at any time, if the investigator deems it in the participant’s best interest. The reason and circumstances for study discontinuation will be documented in the participant’s case report form (CRF). Any subject who discontinues participation and has been treated according to the study protocol should undergo a final examination if possible. The result of the final examination will be documented in the CRF and the data analyzed according to the intention-to-treat principle.

### Follow-up

The duration of ICU and hospital stay as well as the discharge destination are recorded. Patients will be contacted by telephone to assess their quality of life during the follow-up period at 28 days, 90 days and 6 months.

### Study endpoints

The primary endpoint is weaning time, defined as the time from randomization to successful extubation.

Secondary endpoints are: total time of mechanical ventilation; time from randomization to first extubation; time from randomization to fulfillment of extubation criteria; time between fulfillment of extubation criteria and extubation; ICU length of stay, in-hospital length of stay; ICU and in-hospital-mortality; mortality at day 90 and 6 months; PaO_2_/FiO_2_; PaCO_2_; total minutes of ventilation; mean tidal volume; mean pressure support; mean airway pressure; mean peak inspiratory pressure; inspiratory airway occlusion pressure at 100 ms; visual analogue scale for breathing comfort; cumulative amount of sedative and analgesic drugs; SOFA score; re-intubation rate; use of non-invasive ventilation; post-extubation duration of NIV; ventilator-free days (28 days); number of changes in mean pressure support by ICU personnel; coefficients of variation of tidal volume and pressure support; respiratory rate, and inspiratory and expiratory times.

### Statistical considerations

#### Sample size calculation

The sample size calculation (GPower software version 3.1.3, University of Düsseldorf, Germany) was based on the Wilcoxon–Mann–Whitney test (two groups, two tailed). The calculation was based on the expectation that the primary endpoint variable (time from randomization to successful extubation) would show a difference of 2 days between the two treatment groups. Based on the study by Lellouche *et al*. [14] the standard deviation (SD) was estimated as 5 days for both groups, yielding an effect size (*d*) of 0.4. Accordingly, 104 patients per group would allow the detection of differences between the groups with a power of 80% and a type one error rate of 5%. Assuming that 10 dropouts per group may occur, 228 patients in total are necessary.

#### Statistical analysis

The descriptive analysis will include the mean and standard deviation for normally distributed variables. Variables that are not normally distributed will be expressed by their medians and interquartile ranges. Categorical variables will be expressed as *n* (%). Differences in the primary endpoint variable, time from randomization to successful extubation between the two treatment groups, will be analyzed by a Wilcoxon–Mann–Whitney test.

To decide on the proper comparison method for the exploratory analysis of the secondary endpoints, we will test whether the variables are normally distributed. Student’s *t*-test will be used to test groups of independent continuous normally distributed variables. The Mann–Whitney *U* test will be used for continuous variables that are not normally distributed. Categorical variables will be compared with the chi-square test or Fisher’s exact test. Paired data will be analyzed using Student’s *t*-test for continuous normally distributed variables, the Wilcoxon test for continuous variables that are not normally distributed and the McNemar or Bowker test, as appropriate, for categorical variables.

Kaplan–Meier survival curves will be computed to evaluate differences in overall survival. Survival curves will be compared by a log-rank test and multivariable analysis will be accomplished by the Cox regression model.

If any patients are lost to follow-up or withdraw consent for the trial, the reasons will be reported. The intention-to-treat (ITT) analysis and per protocol (PP) analysis will be conducted to test whether the result of this trial is reliable. Missing data will be handled by means of the last-observation-carried-forward method. For the intention-to-treat analysis, data will be processed for all trial patients in the groups to which they were randomized, regardless of whether they received or adhered to the allocated intervention.

It is assumed that the majority of patients in the two triage arms will receive the appropriate study intervention. The per protocol analysis will be performed as a secondary analysis if there are sufficient patients in the triage arms who do not receive study therapy or are lost to outcome assessment. Data from participants who do not violate the treatment protocols will be included in the per-protocol analysis.

### Study organization

The trial is investigator initiated and controlled by a steering committee including anesthetists and intensive care physicians, all with solid research experience. The executive committee comprises the study’s principal investigator and the principal investigators of the investigating centers that approved the final trial design and protocol issued to the clinical sites.

An independent data and safety monitoring board (DSMB) monitors patient safety and reviews safety issues as the study progresses. All serious adverse events and all unexpected and related or possibly related adverse events are reported to the appointed international manager for serious adverse events, who assesses the events and reports this information to the DSMB.

Data management is performed by the Centre for Clinical Studies at Dresden University of Technology, Germany, using study software MACRO 3.0. The data are proven by programmed range checks, validity checks and consistency checks. In addition, there is a manual and visual check of the data for medicinal plausibility according to good clinical practice guidelines. Intervention period data are checked periodically to monitor adherence to the protocol, thereby giving feedback to staff. Trial updates are published on the trial website.

## Discussion

To our knowledge, the present study protocol is the first to address the effects of variable PSV on the duration of weaning in intensive care unit patients. This study is the first international multicenter trial on variable ventilation.

There is a considerable body of experimental evidence showing that variable mechanical ventilation is beneficial in terms of lung function and damage, compared to non-variable mechanical ventilation. Variable controlled ventilation has been shown to improve gas exchange and lung mechanics in experimental models of the acute respiratory distress syndrome (ARDS) in small [15-17] and large animals [11,12,18,19]. Also, variable controlled mechanical ventilation has the potential to reduce histologic lung damage and pulmonary inflammation [18]. The exact mechanisms behind such effects have not been elucidated so far, but release of a lung surfactant [16,20], lung recruitment [21,22] and stochastic resonance [15,19,23] have been suggested. In addition, one randomized controlled trial suggested that intraoperative variable controlled mechanical ventilation improves oxygenation and respiratory system compliance in patients submitted for surgical repair of an aortic aneurysm [24]. However, clinical outcome variables were not reported for that study. Currently, in the PROVAR trial (protective variable ventilation for open abdominal surgery; ClinicalTrials.gov identifier: NCT01683578), our group is investigating whether variable volume controlled ventilation is able to reduce the incidence of postoperative pulmonary complications. Nevertheless, that trial is being conducted in the operation room only. Thus, patients are under general anesthesia and muscle paralysis. In addition, the duration of the intervention is limited to three to ten hours.

Variable PSV is a new mode of mechanical ventilation able to combine variable ventilation with assisted mechanical ventilation. Experimental studies on models of ARDS have demonstrated that variable PSV improves gas, reduces the elastance of the respiratory system and the inspiratory effort as well as respiratory drive, compared to non-variable modes of assisted ventilation. Furthermore, variable PSV reduced lung inflammation and damage in experimental ARDS compared to protective controlled mechanical ventilation [12,18].

The ViPS trial will broaden our knowledge of the impact of variable PSV on important clinical outcome variables in a mixed ICU population. The inclusion and exclusion criteria have been carefully designed to include a large number of patients, regardless of their co-morbidities, while taking safety issues into account. Thereby, the use of variable PSV, which has been evaluated only for hypoxemic lung failure, will be expanded to other patient groups. The protocol described here represents a consensus among centers and investigators with considerable experience in weaning patients from mechanical ventilation. The interventions are an agreement between several weaning strategies conducted in the participating centers, while the variable PSV settings have been carefully derived from experimental knowledge combined with clinical experience. Therefore, compliance of the participating centers to the protocol is likely.

The ViPS trial protocol has limitations that must be addressed. First, the entry criteria allow for patients with large differences in weaning duration, ranging from hours to days. These differences have been considered in the sample size calculation. Second, patients who fail to wean may be transferred to weaning centers, resulting in them being lost to follow-up and, consequently, causing a reduction in study power. Nevertheless, the dropout rates considered in the sample size calculation represent a worst case scenario for the participating centers. Third, the protocol cannot be conducted in a blinded fashion, which can favor bias. To minimize this effect, we defined a tight protocol where intervention steps mandate handling until the primary endpoint is achieved. Fourth, even though the protocol interventions represent a consensus among centers, local differences in weaning experience and standards may still affect the results of this trial. Fifth, the use of non-invasive ventilation following extubation could introduce bias in terms of a reduction of weaning time, but this effect should not affect the comparative performance of variable PSV, since it is also allowed in the control group.

In conclusion, ViPS is the first randomized controlled trial that compares variable with non-variable PSV with respect to the duration of weaning from mechanical ventilation in a mixed ICU population. This trial may also clarify how and why variable PSV improves lung functional variables, and reduces the respiratory drive.

### Trial status

Participating centers:

•University Hospital Dresden, Dresden, Germany

•Hospital de la Santa Creu i Sant Pau, Barcelona, Spain

•University Hospital in Reus, Spain

•Medical University of Vienna, Vienna, Austria

•IRCCS San Martino Hospital, University of Genoa, Italy

•Academic Medical Center at the University of Amsterdam, Amsterdam, The Netherlands

Enrollment is expected to be completed in the second quarter of 2015. The end of the study will be in the fourth quarter of 2015.

## Abbreviations

APACHE: Acute physiology and chronic health evaluation (score); ARDS: Acute respiratory distress syndrome; BPS: Behavioral pain scale; CONSORT: Consolidated standards of reporting trials; CPAP: Continuous positive airway pressure; CRF: Case report form; DSMB: Data and safety monitoring board; FiO2: Inspiratory oxygen fraction; ITT: intention-to-treat; ICU: Intensive care unit; NIV: Non-invasive ventilation; P0.1: Airway inspiratory occlusion pressure at 100 ms; PaO2: Arterial partial pressure of oxygen; PaCO2: Arterial partial pressure of carbon dioxide; PEEP: Positive end-expiratory pressure; PP: Per protocol; PSV: Pressure support ventilation; RASS: Richmond agitation and sedation scale; RR: Respiratory rate; SaO2: Oxygen saturation; SOFA: Sequential organ failure assessment (score); SSL: Secure sockets layer; VAS: Visual analogue scale; ViPS: Variable pressure support trial; VT: Tidal volume.

## Competing interests

MGA, PMS and TK have been granted a patent for the noisy or variable pressure support ventilation type of assisted mechanical ventilation. Dräger Medical AG (Lübeck, Germany) provided a restricted research grant (20,000 euros) to conduct the study and gave each participating site one or two mechanical ventilators able to deliver variable PSV for the duration of the trial.

## Authors’ contributions

TK is the study coordinator and participated in the design of the study, performed the statistical analysis and drafted the manuscript. AG and TB participated in the design, statistical analysis plan and even drafting of the manuscript. They did not contribute equally as the first author. CU and BW participated in designing the study, contributed to the statistical analysis and drafted the manuscript. PMS participated in designing the study, contributed to the statistical analysis and contributed to drafting the manuscript. KM, RU, SJ, JM and PP participated in designing the study and drafted the manuscript. LC drafted the manuscript. JAS, RB, GS, FS, IV and TK contributed to drafting the manuscript. MGA is the principal investigator, participated in designing the study, contributed to the statistical analysis and drafted the manuscript. All authors read and approved the final manuscript.
